# Physiological, antimicrobial, intestine morphological, and immunological effects of fructooligosaccharides in pigs

**DOI:** 10.5194/aab-63-325-2020

**Published:** 2020-09-10

**Authors:** Brigitta Csernus, Levente Czeglédi

**Affiliations:** 1 Department of Animal Science, Institute of Animal Science, Biotechnology and Nature Conservation, Faculty of Agricultural and Food Sciences and Environmental Management, University of Debrecen, Debrecen, 4032, Hungary; 2 Doctoral School of Animal Science, University of Debrecen, Debrecen, 4032, Hungary

## Abstract

In pig nutrition, there are some periods when natural alternatives to antibiotics are more required, such as during suckling and weaning. Fructooligosaccharides (FOSs) are a group of prebiotics applied as feed ingredients in animal nutrition since their positive effects on growth performance, immunological parameters, intestinal microbiota, and gut morphology are reported. Accordingly, FOS may be candidate molecules to improve the mentioned properties in pigs. Previous studies defined FOS as inhibiting the activity of pathogens and increasing the colonization of beneficial bacteria in the gut, although metabolites of FOS decreased the intestinal pH value. Beneficial effects on digestive-enzyme activities and on protein digestion were determined in some studies. All of the three types of FOS (inulin, oligomeric fructans, and short-chain FOSs) promoted the microbial composition of the gut by increasing the colonizations of *Lactobacillus*, *Bifidobacterium*, and *Prevotella* genus. FOS also affected the immune response directly and indirectly and increased vaccine-specific IgA, serum IgG, and IgE levels. Moreover, FOS enhanced the activation of T cells and altered the secretions of some cytokines. Levels of vaccine-specific IgG could not be increased after FOS supplements. In most cases, FOS modified intestinal morphological parameters, such as longer villi, villus-height-to-crypt-depth ratio, and thicker mucosa, which could suggest better absorptive functions. Results are contradictory on growth performance, which might be influenced by the chemical structure, the duration, and the dose of FOS, so further studies are required. This review aims to gather information regarding immunological, antimicrobial, intestine morphological, and growth performance properties of fructooligosaccharides in pigs.

## 1 Introduction

EU legislation prohibits the use of feed antibiotics; therefore, it is necessary to find alternatives which aim to maintain gut health in pigs (Loh et al., 2006; Verdonk et al., 2005). Frequently, antibiotics have been substituted with nutritional alternatives in recent years (Yang et al., 2009; Khodambashi Emami et al., 2012). For pigs, substitution is more required since they are threatened in critical periods, such as suckling or weaning. In the early postnatal period of pigs, gut-associated lymphoid tissues (GALTs) are undeveloped and microbial colonization in the gut does not occur. At weaning, alteration in feed and environment causes a stressful lifetime and results in a microbial imbalance which induces a lower immune response, more sensitiveness to illness, and a loss in production performance (Inman et al., 2005; Jacela et al., 2010). Accordingly, it is necessary to improve intestinal health and performance. Several herbs and feed additives contain important bioactive compounds, the effects of which are often investigated with regard to the physiology of livestock species, including porcine ones (Demir et al., 2010; Flickinger et al., 2003). In most cases, feed ingredients target the microbiota of the gastrointestinal tract, since its composition correlates with health status, nutrient absorption, and weight gain (Yang et al., 2009; Barrow, 1992). One of the possible natural alternatives is prebiotics, i.e., indigestible, fermentable feed additives which enhance the growth of beneficial bacteria, such as *Lactobacillus* and *Bifidobacterium*, which can prevent diarrhoea and support intestinal integrity (Gibson and Roberfroid, 1995; Gibson et al., 2004; Zhao et al., 2019). Improved intestinal integrity can help to increase the colonization of *Prevotella* spp., which maintain intestinal homeostasis (Zhao et al., 2019). Fructooligosaccharides (FOSs) can reduce harmful pathogens of the microbiome as well, therefore resulting in a better health status of the host (Salminen et al., 1998; Micciche et al., 2018). FOSs are a well-known group of prebiotics (Gibson and Roberfroid, 1995). In addition to their prebiotic effects, they are also applied as food ingredients or functional food (Gibson and Roberfroid, 1995; Singh et al., 2016). They are non-digestible oligosaccharides consisting of β-(2-1)-linked fructosyl units with a glucose unit as a terminate (Roberfroid et al., 2010). The appearance of FOS takes three forms: inulin, oligofructose, and short-chain fructooligosaccharides (scFOSs) (Roberfroid and Delzenne, 1998). FOS can support the microbiota in the intestine of pigs, which is consistent with the status of the immune system (Shim et al., 2005; Babu et al., 2012). These prebiotics are useful as anti-inflammatory agents, as they can boost both innate and acquired immune responses (Khodambashi Emami et al., 2012). As well as immunological effects, microbial fermentation enhances the absorption of some minerals, such as calcium, magnesium, and iron (Yu Wang et al., 2010). In addition, FOS have the potential to improve mucosa structurally (Xu et al., 2003). Equally important, production performance in porcine animals may be higher after FOS supplementation (Estrada et al., 2001; He et al., 2002; Chang et al., 2018).

## 2 Chemical structure and sources of fructooligosaccharides

Fructooligosaccharides are classified as a group of inulin-type oligosaccharides known as fructans and consist of 2 to 60 D-fructose units with β-(2,1) linkages (Apolinário et al., 2014; Gibson and Roberfroid, 1995; Sabater-Molina et al., 2009). They are naturally occurring short-chain carbohydrates with a D glucosyl ending and are widely found in plants such as chicory root, onion, garlic, beet, cane sugar, asparagus, artichoke, banana, wheat, and yacón root (Micciche et al., 2018; Yun, 1996; Vandeplas et al., 2009; Caetano et al., 2016; Williams et al., 2009). There are three types of fructooligosaccharides which have variant polymerization degrees, and they are also different in origin and structure. Linear long-chain fructans are mostly extracted from chicory roots and are called inulin, which is usually composed of 12 fructose units. Oligomeric fructans (degree of polymerization from 3 to 9), usually called oligofructose or fructooligosaccharides, are mostly obtained from the hydrolysis of inulin or enzymatic synthesis from sucrose (beet or cane). Oligofructose consists of fructosyl chains and end in fructose or glucose monomers. Short-chain fructooligosaccharides (scFOS) are the third type of fructooligosaccharides and consist of different fructosyl chains with a glucose monomer at the end (Roberfroid and Delzenne, 1998; Roberfroid, 2005). Based on β-(2,1) linkages, fructooligosaccharides are difficult to be digested by small intestinal enzymes, so they reach the large intestine and are degraded by intestinal bacteria (Langen and Dieleman, 2009). Fructooligosaccharides may be solubilized in water, and the sweetness of FOS is 0.3–0.6 fold higher than the sweetness of sucrose, which is impacted by the polymerization degree of oligosaccharides (Crittenden and Playne, 1996; Yun, 1996). Water-holding capacity and thermal stability are also high in FOS, and they are stable in a pH range of 4 to 7 (Yun, 1996; Mussatto and Mancilha, 2007). FOS also have properties such as low sweetness intensity and being calorie-free and non-cariogenic (Yun, 1996; Rivero-Urgell and Santamaria-Orleans, 2001).

## 3 Effect on digestion

Fructooligosaccharides (Meiji Seika Kaisya, Ltd., Tokyo, Japan; 97.2 % FOS content; GF${}_{2}$, GF${}_{3}$, and GF${}_{4}$ amount to 46.8 %, 39.3 %, and 11.1 %, respectively) at 4 and 6 g kg${}^{-1}$ in the diet supported the activities of digestive enzymes such as total protease, trypsin, and amylase measured in the small intestinal contents of growing barrows at experimental day 42 (Xu et al., 2002). These changes may contribute to the altered microbial ecosystem and the presence of FOS which stimulates *Lactobacillus* and *Bifidobacterium*, i.e., those bacteria which colonize in the intestine, transfer enzymes, and enhance digestive-enzyme activities (Sissons, 1989). In the same study, FOS did not affect chymotrypsin, lipase, or any of the digestive-enzyme activities in the pancreas (Xu et al., 2002). Shim (2005) reported that neither 0.25 % nor 3 % FOS affected maltase, lactase, or sucrose activities in the duodenum, jejunum, or ileum in male pigs (weaned at 24.2 d old) at the end of a 3-week feeding trial. However, enzyme activities tended to increase in the duodenal site of the small intestine in FOS-treated pigs, a phenomenon which may be caused by the increased feed intake and the higher disaccharide content, since the activity of these enzymes depends on the presence of substrates. The activity of disaccharidases is important in the enzymatic breakdown of carbohydrates, which influence nutritional processes (Shim, 2005). In the same study, the digestion of crude protein was higher in FOS-treated pigs compared to the control: digestibility was 75.8 %, 72.5 %, and 68.4 % in pigs fed with a FOS-supplemented diet at 0.25 %, 3 %, and 0 %, respectively. Ileal digestion of protein indicates the utilization of amino acids through the intestinal villi.

The effect of FOS on digestion is different among experiments, which may be related to the alterations in the microbial ecosystems, disaccharidase contents of the diet, and the presence of different substrates.

## 4 Gut microbiota composition

Intestinal microbial fermentation is beneficial for the activity of the microbial population: *Lactobacillus* and *Bifidobacterium* have the ability to ferment fructooligosaccharides, but less is known about which strains could ferment these oligosaccharides. Kaplan and Hutkins (2000) identified *Lactobacillus acidophilus *strains, especially DDS-1 and NCFM, *L. plantarum* MR240, *L. casei* MR191, and 685. *Bifidobecterium adolescentis*, *B. breve*, *B. infantis*, and *B. longum* are those able to metabolize fructooligosaccharides. In porcine animals, FOSs are metabolized mainly in the cecum and colon but also in the stomach or ileum harbor (Conway, 1994; Jensen and Jorgensen, 1994). They are fermented into several metabolites, mainly short-chain fatty acids (SCFAs). FOS fermentation results in the stimulation of the activities of probiotic bacteria, such as *Lactobacillus* and *Bifidobacterium*, which are part of the intestinal commensal microbiota (Salminen et al., 1998; Macfarlane and Cummings, 1999; Boguslawska-Tryk et al., 2012). Consequently, cecal SCFA and lactate content increase, thereby inhibiting the activity of harmful bacteria such as *Escherichia coli *or *Salmonella* species in the gut (Hidaka et al., 1986; Choi et al., 1994; Bunce et al., 1995; Roberfroid et al., 1998; Fukata, 1999; Xu et al., 2002). Xu et al. (2002) confirmed the same when 4 and 6 g kg${}^{-1}$ FOS (type is described in Sect. 3) supplementation in the diet of growing barrows (average body weight of 20.8 kg) increased the colonization of *Lactobacillus* and *Bifidobacterium* and decreased the cell numbers of *Clostridium* in the small intestine at the end of the experiment (day 42). In the proximal colonic content, cell counts of the beneficial bacteria were increased similarly by 4 and 6 g kg${}^{-1}$ FOS addition, and colonization of *Clostridium* was already decreased in pigs fed a 2 g kg${}^{-1}$ FOS-supplemented diet, and cell numbers of *Escherichia coli *were smaller in 4 g kg${}^{-1}$ FOS-treated barrows. Shim (2005) determined that *Bifidobacterium* cell numbers increased in the terminal ileum of 21 d old piglets and the numbers of *E. coli* also decreased in the colon when the diet contained 0.2 % oligofructose from 7 to 21 d of age. In the same experiment, the number of *Lactobacillus* *acidophilus* did not change in either the ileum or the colon. Longer-chain fructans increased *Lactobacillus* and decreased *E. coli* cell numbers in the feces of finishing pigs at the end of week 6 when 1 % and 2 % fructan was added to their diet (Zhao et al., 2013). Microbiota composition could be modified in 21 d old suckling piglets when the maternal diet was supplemented with scFOS (95 % scFOS with molecular chain length between 3 and 5 monomeric units) at 3.3 g kg${}^{-1}$ during the last 4 weeks of gestation and 1.5 g kg${}^{-1}$ during the 4 weeks of lactation, as well. The relative proportion of bacteria from the Bacteroidetes phylum could increase, while the relative abundance of bacteria from the Firmicutes phylum decreased. At the genus level, *Prevotella*, an unclassified genus belonging to the Bacteroidales order, and the *Treponema* genus increased, while the *Bacteroides* genus and an unclassified genus belonging to the *Ruminococcaceae* family were reduced after maternal diet supplementation. In the same study, a high proportion of the Bacteroidetes phylum, especially the *Prevotella* genus, could be maintained in older pigs (at postnatal day 190) as well. In addition, the proportion of an unclassified genus belonging to the *Lachnospiraceae* family was higher and decreased the unclassified genus belonging to the Bacteroidales order, and an unclassified genus belonging to the *Ruminococcaceae* family was identified on the same experimental day. *Prevotella* genus abundance was still higher and the proportion of the Proteobacteria phylum was decreased at postnatal day 211. (Le Bourgot et al., 2014; Le Bourgot et al., 2019). ScFOS at 4 g kg${}^{-1}$ in the diet could also alter gut microbiota colonization in the colonic chyme of male piglets, such as an increased relative abundance of the Bacteroidetes phylum, *Lactobacillus* spp., *Bifidobacterium* spp., and *Prevotella* spp. when the feeding trial was started at 28 d of age and lasted for 28 d (Zhao et al., 2019).

However, populations of *Lactobacillus* and *Bifidobacterium* did not change in 42 d old castrated growing pigs when the diet included 10 g kg${}^{-1}$ FOS (Raftifeed^®^, OPS, Orafti, Belgium) for 32 days (Mountzouris et al., 2006). A number of *Lactobacillus* and *Bifidobacterium* were not stimulated by inulin at 3 % in the diet fed for 3 and 6 weeks in the intestinal contents of castrated pigs when the experimental period was started on the 42nd day of life (Loh et al., 2006). Bacterial composition was not affected when inulin at 3 % for 3 and 6 weeks was used as supplementation starting 5 and 8 weeks after weaning (28 d), either (Eberhard et al., 2007).

In most cases, FOS supplementation increased the colonization of beneficial bacteria, while the cell numbers of harmful ones decreased, which may be due to the higher cecal lactate- and SCFA concentration. All of the three types of FOS (short-chain FOS, oligomeric fructans, and longer-chain fructans) could aid beneficial microbial composition, since they could promote the colonizations of *Lactobacillus*, *Bifidobacterium*, or *Prevotella* genus and decrease the harmful bacteria, such as *Clostridium* or *Escherichia coli*, in most cases. The dosage and duration of the different types of FOS may be influencing factors as well.

## 5 Metabolism of fructooligosaccharides and effects on intestinal pH value

Fructooligosaccharides cannot be digested in the upper gastrointestinal tract because of the glycosidic linkages in the molecule which contain β conformation of the C2 atom in fructose units (Gibson and Roberfroid, 2008). They are hydrolyzed by the intestinal bacteria since they produce enzymes, such as fructan-β-fructosidase or exo-inulase (Wang and Gibson, 1993). Fructooligosaccharides are fermented into several metabolites, such as SCFAs: primarily acetate, propionate and butyrate (Cummings and Englyst, 1995; Van Loo et al., 1999). Besides SCFAs, prebiotics are also converted to biomass and gases, such as hydrogen, carbon dioxide, and methane (Van Loo et al., 1999; Ohta et al., 1995). SCFAs can be absorbed rapidly and they have roles in biological processes. SCFAs, such as acetate and propionate, are bacterial metabolites and promote the release of glucagon-like peptide-1 (GLP-1) by distal small intestinal and proximal colonic L cells since SCFAs activate the G-protein-coupled free fatty-acid receptor 2 (Tolhurst et al., 2012). GLP-1, namely incretin, induces B-cell mass and insulin secretion capacity (Baggio and Drucker, 2007). This gut–pancreas communication is important for maintaining glucose homeostasis integrated during the enteroinsular axis. Gut microbial composition is responsible for enteroinsular communication, so it influences the glucose homeostasis (De Filippo et al., 2010; Dominguez-Bello et al., 2010; Penders et al., 2006). Butyrate is important for the energy metabolism in large intestinal mucosa since it stimulates the growth of epithelial cells (Roediger, 1980). Other fatty acids, including propionate or butyrate, also serve as substrates for the liver, and acetate is metabolized by the muscle and peripheral tissues (Bergman, 1990; Salminen et al., 1998; Blaut, 2002). Since acetate is strongly acidic, it provides a low pH value which decreases the colonization of pathogens (Sabater-Molina et al., 2009). For example, Houdijk et al. (2002) measured a lower pH value in the ileum of weaner castrated pigs supplemented by 40 g kg${}^{-1}$ FOS (Raftilose P95^®^ powder with 90 % FOS) in the diet for 13 d, and a feeding trial was started at the age of 38 d. In the same study, the concentration of acetic acid was lower, and propionic acid and lactic acid concentrations were higher than in control pigs. Isovaleric acid was also present at a higher concentration and may denote intensified FOS fermentation. Shim et al. (2005) also measured lower pH values in the cecum and proximal colon, after FOS (Neosugar^®^, on a dry-matter basis was 95 % dry matter, with 50 % of FOS with a terminal glucose and three fructose units) at 3 % was supplemented for 21 d in 24 d old weaning pigs. This lower pH value may be parallel to the increased volatile fatty-acid (VFA) content, mainly acetate and lactate (Gibson and Roberfroid, 1995). In the same study, concentrations of butyrate and isobutyrate decreased after 0.25 % FOS supplementation; the concentration of valerate was lowered to a 3 % FOS addition. The contents of acetate and isovalerate also decreased after both concentrations of FOS. A lower intestinal pH value supports the activity of *Bifidobacterium* but inhibits the growth of harmful bacteria, such as *Escherichia coli* (Shim et al., 2005). An acidic environment is also necessary for effective mineral solubilization. Previous studies with rats mentioned oligofructose (Raftilose^®^ P95 with 950 g kg${}^{-1}$ oligofructose is made with enzymatic hydrolysis of chicory inulin, with a degree of polymerization ranging from 3 to 7, average of 4) and long-chain inulin (Raftiline^®^ HP with 955 g kg${}^{-1}$ long-chain chicory inulin with a degree of polymerization ranging from 10 to 60, average of 25) supplementation at 25 g kg${}^{-1}$ during the adaptation phase (7 d) and at 50 g kg${}^{-1}$ during the experimental phase (8 d); increased cecal contents (Kleesen et al., 2001) and FOS (NutraFlora^®^) or oligofructose (Raftilose^®^) at 6 % in the diet increased cecal wall weight and cecal total weight, which ensures a higher volume for mineral absorption (Campbell et al., 1997). Although Tsukahara et al. (2003) reported that the pH value was not changed in digesta of 47 d old pigs after 10 % FOS (Meiji Seika Kaisya, Ltd., Tokyo, Japan) supplementation for 10 d, the concentration of n-butyrate was increased in the cecum and centripetal gyri and n-valerate also tended to be higher.

Accordingly, the authors determined that FOS decreased the pH value in most cases since the concentration of lactate or lactic acid was increased. The effect of FOS on the pH value may depend on the type of FOS such as the degree of polymerization, the structure, the origin, the dose, and the duration of supplementation.

## 6 Intestinal morphological effects

Morphological changes in the intestine can reveal the health status of the gut. Stress factors can modify the mucosa layer rapidly, since the digesta content and the mucosal surface are so close to each other. Accordingly, smaller villus length and deeper crypt depth may indicate toxin occurrence (Yason et al., 1987). The health of the small intestinal surface is related to the appropriate absorption of nutrients. Therefore, changes in the intestinal area, including greater villus length, decreased crypt depth, and thicker mucosa, can influence absorption positively (Shang et al., 2015). In some studies, fructooligosaccharides modified these intestinal morphological parameters. Fructooligosaccharide (FOS contained 1.83 % monosaccharides, 1.13 % sucrose, 39.81 % kestose, 49.78 % nystose, and 7.06 % furanosyl nystose for a total FOS of 96.65 %) at 0.4 % in the diet increased the villus length of the jejunal and ileal mucosa in mixed-sex pigs when the experiment lasted for 21 d (Xu et al., 2005). Similarly, FOS at 4 or 6 g kg${}^{-1}$ in the diet of growing barrows induced a higher jejunal villus length, and the villus height : crypt depth ratio in the mucosal layer contrasted with the control group at the end of the experiment (day 42) (Xu et al., 2002). Tsukahara et al. (2003) determined thicker mucosa in the hindgut of 47 d old weaning piglets, when the diet was supplemented with 10 % FOS for 10 d. Le Bourgot et al. (2017) measured higher empty cecum weight in weaning pigs at postnatal day 56 when the maternal diet was supplemented with scFOS (in the same way as described in Sect. 4) compared to the control maternal diet group. The empty colon weight and the number of goblet cells in a crypt in the cecum also tended to increase with maternal scFOS supplementation. In contrast, relative small intestinal length and mucosa weight, ileal villus length, and crypt depth did not differ in scFOS supplemented piglets compared to control ones (Le Bourgot et al., 2014). Villus heights were not altered in the duodenum or ileum of weaning piglets, when the diet contained 0.25 % oligofructose (Shim, 2005).

The effect of FOS on intestinal morphological parameters was mostly positive and FOS could improve villus length, mucosa thickness, and empty cecum weight. A beneficial effect was shown by a concentration of 0.4 % and positive changes were determined in mixed-sex pigs or barrows. FOS could also alter a morphometric parameter in the intestine indirectly when piglets' mothers were fed scFOS supplementation during the last 4 weeks of gestation and the 4-week lactation periods. Impacts of the different types of FOS were contradictory: oligomeric fructans (FOS) could promote longer villi and thicker mucosa, although villi length was not affected in another study; short-chain FOS could promote higher empty cecum weight, but villus length, crypt depth, and relative small intestinal length were not affected in any other experiment.

## 7 Immunological activity

Fructooligosaccharides can affect immune responses indirectly (Caetano et al., 2016). Microbial fermentation of FOS results in SCFA production, which changes the microbiota in the intestine and influences immune responses (Caetano et al., 2016; Babu et al., 2012). SCFAs can control interleukin production and natural killer cell (NK cell) activity (Kim et al., 2014). FOS can also stimulate immune responses directly, through GALT (Peshev and Van den Ende, 2014). Molecules of fructans or polysaccharides may enhance the activation of specific cells of the immune system, as macrophages, dendritic cells, lymphocytes, or neutrophils, because pathogen-associated molecular patterns (PAMPs) are bonded with toll-like receptors (TLRs), which result in immunostimulation (Liu et al., 2011). For example, a yacón (which contains FOS) treatment increased TLR-4 expression in baby mice (Delgado et al., 2012; Velez et al., 2013). Levels of total immunoglobulin G and immunoglobulin E in serum were also increased in 32 d old barrows fed with a 0.6 % FOS (Solarbio, Beijing, purity >95 %) supplemented diet for 11 d compared to control pigs (Chang et al., 2018). Le Bourgot et al. (2014) measured higher serum IgA levels in sows between the 87th day and the 108th day of gestation when the diet was supplemented with 3.3 g kg${}^{-1}$ short-chain fructooligosaccharides (scFOS 95 % with molecular chain length between 3 and 5 monomeric unity; Beghin-Meiji, Marckolsheim, France) during the last 4 weeks of gestation and at 1.5 g kg${}^{-1}$ during the 4 weeks of lactation. The level of serum IgG did not change in the scFOS-supplemented group (Le Bourgot et al., 2014). A specific serum IgA response to the influenza vaccine could increase in sows after weaning when the diet involved scFOS at 0.15 % (95 % of scFOS with a molecular chain length between 3 and 5 monomeric unity; Profeed P95^®^ Beghin-Meiji, Marckolsheim, France) during the post-weaning period and also when the maternal diet involved scFOS at 3.3 g kg${}^{-1}$ during gestation and 1.5 g kg${}^{-1}$ during lactation. In the same study, the specific influenza IgA level was also increased in the feces of weaning pigs at day 77 after diet was supplemented with scFOS at a concentration of 0.15 %. Vaccine-specific IgG levels were not affected by maternal or pig scFOS supplementation (Le Bourgot et al., 2016). In another study, a vaccine-specific IgA level could increase in serum and tended to increase in ileal mucosa of piglets at postnatal day 54 when the same maternal scFOS diet was applied. A positive correlation was also determined between total sIgA and vaccine-specific IgA in the ileal mucosa (p<0.001, R=0.84). Vaccine-specific IgG was not influenced in serum at postnatal day 56 in this study either (Le Bourgot et al., 2017). The addition of scFOS to the maternal diet did not influence serum IgG and IgA in piglets between postnatal days 7 and 21, compared to the control piglets. In addition, levels of some cytokines were measured and the secretion of interferon (IFN) γ was higher in jejunal and ileal Peyer's patches and in mesenteric lymph node cells of piglets at postnatal day 21 when scFOS supplementation was applied in the maternal diet in the same way. The concentrations of secreted interleukin (IL) 10 and tumor necrosis factor (TNF) α did not differ between groups in the same study (Le Bourgot et al., 2014). The density of mesenteric lymph node cells was also higher in piglets when maternal feed contained scFOS, and this could be considered to be a sign of a stimulated immune cell function of the intestinal immune system development. The secretion of IgA was also enhanced in ileal Peyer's patches and tended to be enhanced in the jejunal Peyer's patches of scFOS treated pigs. Equally important, scFOS-treated pigs showed an increased proportion of activated CD25+CD4α + T cells among T helper cells which was in positive correlation with the secreted IFN-γ level in ileal Peyer's patch cells (p<0.05, Pearson $R^{2}=0.29$). This may signal more active CD4${}^{+}$ T cells and a better Th1 response polarization of Peyer's patches and in mesenteric lymph node cells and thereby a more effective immunity against pathogens. The level of haptoglobins in blood plasma was also increased at postnatal day 21 when the scFOS maternal diet was applied, and it was discussed in connection with the enhanced cellular immune response of Th1 cells (Le Bourgot et al., 2014). In another study, TNF-α expression could decrease in the visceral adipose tissue of piglets when the maternal diet was supplemented with scFOS at 3.3 g kg${}^{-1}$ during the last 4 weeks of gestation and 1.5 g kg${}^{-1}$ during the 4 weeks of lactation (Le Bourgot et al., 2019). The secretion of IFN γ was improved and interleukin-4 (IL-4) tended to increase in the ileal mucosa of pigs at postnatal day 56 after maternal scFOS supplementation. Short-chain FOS supplementation in the post-weaning diet at 0.15 % tended to result in a lower concentration of ileal TNF α in pigs (Le Bourgot et al., 2017). Immune factors, such as lymphocytes, leukocytes, neutrophils, and CD4${}^{+}$ T cells tended to be enhanced when a combination of FOS (Raftilose P95^®^) at 3 g per animal per day and *Lactobacillus* *paracasei* (1.10${}^{9}$ CFU g${}^{-1}$) in powder milk at a dose of 2 g per animal per day were applied for 10 d after birth (Herich et al., 2002). Blood lymphocyte and neutrophil concentrations were measured in another study, and higher concentrations were expected as a sign of enhanced immune response, though concentrations were not changed in 46 d old weaning piglets after 0.2 % oligofructose (95 % dry matter (GF${}_{2}$ : 1-kestose 35 %, GF${}_{3}$ : nystose 50 %, and GF${}_{4}$ : 1-fructosyl-nystose 10 %) + 5 % of glucose + fructose + sucrose) supplementation for 21 d (Shim, 2005).

Accordingly, FOS could affect the immune response directly through enhancing the levels of IgE and IgG in the serum of barrows, even at a concentration of 0.6 % in the diet. Short-chain FOS could also enhance immunity both directly and indirectly by increasing the levels of vaccine-specific IgA and other immunoglobulins, such as IgA, IgG, and IgE in serum. Immunological activity was further increased indirectly when the scFOS addition altered the levels of some cytokines, and IFN γ was higher and the expression level of TNF α was lower or tended to be lower in piglets. scFOS supplementation induced a higher proportion of CD25 + CD4α + T cells in piglets directly and haptoglobin levels were also observed after maternal scFOS supplementation. Vaccine-specific IgG could not be altered in most cases after scFOS additions.

## 8 Effect on growth performance

Several studies refer to the positive effect of FOS on growth performance of porcine animals. Chang et al. (2018) experienced a higher body weight gain in 32 d old barrows after 0.6 % FOS (Solarbio, Beijing, purity >95 %) supplementation compared to an allergy group, which was sensitized with soybeans. In addition, FOS (97.2% FOS amount) supplementation at 4 and 6 g kg${}^{-1}$ in the diet could increase average daily gain (ADG) and feed conversion ratio in growing barrows compared to the control pigs at the end of the experiment (day 42) (Xu et al., 2002). ADG of porcine animals weaned early was also higher when fructooligosaccharide supplementation at a concentration of 0.5 % was applied for 21 d (Estrada et al., 2001). Le Bourgot et al. (2016) determined increased growth performance of weaned pigs on experimental day 56 and 77 when the maternal diet contained scFOS, as described above. Daily body weight gain was also higher in maternal scFOS supplemented pigs compared to the control over the post-weaning 7 weeks. Shim (2005) determined higher pre-weaning body weight gain when the diet of 46 d old piglets was supplemented with 0.2 % oligofructose for 21 d. Shim also suggests using commercially available FOS products from a concentration of 0.25 % to improve the production performance at weaning. It was also suggested that the effects of FOS may be influenced by several factors, such as the chemical structure of the product, the diet, the age, and the weight of the pig (Shim, 2005). Fructan, which has a longer-chain, could also reach higher ADG and gain : feed (G : F) ratio in 1 % and 2 % fructan supplemented finishing pigs at the end of week 6 (Zhao et al., 2013). ADG of piglets increased during the pre-starter period, when the diet contained the same scFOS at 1.2 g d${}^{-1}$. ADG of piglets also increased during the starter period, when sow diet involved the same scFOS at 10 g d${}^{-1}$. In addition, average daily intake (ADI) decreased during the starter phase when the maternal diet involved the same amount of scFOS. The feed conversion ratio was also improved during the pre-starter and starter phases when both the piglet and maternal diets contained scFOS. Finally, body weight (BW) increased during the pre-growing and growing periods, when piglets received scFOS at 1.2 g d${}^{-1}$ during the pre-starter period (Apper et al., 2016). In contrast with these results, Xu et al. (2005) did not measure significant differences in the weights of pigs fed FOS (contained 1.83 % monosaccharides, 1.13 % sucrose, 39.81 % kestose, 49.78 % nystose, and 7.06 % furanosyl nystose for a total FOS of 96.65 %) at a concentration of 0.4 % for 21 d. However, Xu et al. (2005) noted a trending increase in weight gain. Le Bourgot et al. (2017) did not observe body weight gain in piglets during lactation when the maternal diet was supplemented with scFOS, as described above, and neither maternal diet nor post-weaning diet modified body weight gain at postnatal day 56. Le Bourgot et al. (2014) did not experience enhanced production performance in sows at the end of gestation or during lactation when the maternal diet was supplemented with 3.3 g kg${}^{-1}$ short-chain fructooligosaccharides during gestation and 1.5 g kg${}^{-1}$ short-chain fructooligosaccharides during lactation. In addition, the body weight of sows decreased from day 80 of gestation to day 28 postpartum, and daily feed intake was higher in scFOS supplemented sows at the end of lactation. The BW and ADG of suckling piglets were very similar in the treated and control groups when sow diets contained 10 g d${}^{-1}$ scFOS (Profeed P95, 95 % scFOS with molecular chain length from 3 to 5; 37 % GF${}_{2}$, 53 % GF${}_{3}$, and 10 % GF${}_{4}$) (Apper et al., 2016).

Effects of FOS on growth performance are contradictory. These differing results may be influenced by the different chemical structures and chain length of FOS applied in the studies and the occurrence of other fermentable components in feed, such as non-starch polysaccharides (Shim, 2005). Other studies are required to identify the precise concentration and duration of FOS to improve the growth parameters of pigs.

**Table 1:** Effects of fructooligosaccharides (FOSs) in different chemical structures on reviewed parameters.

Biological activity	Effect${}^{a}$	Structure ofmolecule	Effect mechanism${}^{b}$	References
Positive effect ondigestion	+	oligomeric FOS	direct	Xu et al. (2002), Shim (2005)
Improved gut	+	oligomeric FOS	direct	Xu et al. (2002), Shim (2005)
microbiota		longer-chain FOS	direct	Zhao et al. (2013)
composition		scFOS	direct	Zhao et al. (2019)
		scFOS	indirect	Le Bourgot et al. (2019)
	o	oligomeric FOS	direct	Mountzouris et al. (2006)
		longer-chain FOS	direct	Loh et al. (2006), Eberhard et al. (2007)
Effect on pH value	+	oligomeric FOS	direct	Houdijk et al. (2002), Shim et al. (2005)
	o	oligomeric FOS	direct	Tsukahara et al. (2003)
Positive intestinal	+	oligomeric FOS	direct	Xu et al. (2002, 2005), Tsukahara et al. (2003)
morphologic effects		scFOS	indirect	Le Bourgot et al. (2017)
	o	oligomeric FOS	direct	Shim (2005)
		scFOS	indirect	Le Bourgot et al. (2014)
Immunological activity	+	oligomeric FOS	direct	Chang et al. (2018)
		scFOS	direct	Le Bourgot et al. (2014), Le Bourgot et al. (2016)
		scFOS	indirect	Le Bourgot et al. (2016, 2017, 2019)
	o	oligomeric FOS	direct	Herich et al. (2002), Shim (2005)
Effect on growthperformance	+	oligomeric FOS	direct	Chang et al. (2018), Xu et al. (2002), Estrada et al. (2001), Shim (2005)
		scFOS	direct	Apper et al. (2016)
		scFOS	indirect	Le Bourgot et al. (2016)
		longer-chain FOS	direct	Zhao et al. (2013)
	o	oligomeric FOS	direct	Xu et al. (2005)
		scFOS	direct	Le Bourgot et al. (2017)
			indirect	Le Bourgot et al. (2014, 2017), Apper et al. (2016)

${}^{a}$ +: positive effect on traits/parameters; o: no significant effect was observed. ${}^{b}$ Direct: experiment animals were fed with diet containing FOS; indirect: maternal diet included FOS.

## 9 Conclusions

The effect of FOS with different chemical structures (Table 1) was reviewed in this study. Fructooligosaccharides are inulin-type oligosaccharides consisting of fructose units with β-(2,1) linkages, so they cannot be digested in the small intestine and molecules reach the cecum, where they are fermented by intestinal microbiota. Through these mechanisms, SCFAs and other metabolites are produced. SCFAs provide an acidic environment as they decrease intestinal pH value, which is beneficial for effective mineral solubilization and pathogenic elimination. Prebiotic effects of fructooligosaccharides not only result in a decrease in harmful pathogens, such as *E. coli* in the gut, but all of the three forms (longer-chain fructans, oligomeric fructans, and short-chain fructooligosaccharides) can enhance the activity and growth of beneficial bacteria, such as *Lactobacillus*, *Bifidobacterium*, or *Prevotella*. The effect of FOS on digestion differs, as based on some studies, which might be the result of the altered microbiota in the gut or the presence of different substrates. FOS supplementation – direct and indirect – can promote beneficially changed intestinal morphological parameters which are related to nutrient absorption. The immune response may be affected directly and indirectly as well, by increasing the levels of some immunoglobulins and altering the levels of some cytokines even at a concentration of 0.4 %. The effects of FOS on growth performance are contradictory, which may be due to the differing chemical structures, dosage, and duration of FOS. FOS represents a nutritional strategy of interest in pigs to improve the beneficial bacteria of the microbiota and stimulate their immune system, with beneficial effects on health and growth.

## Data Availability

All data used in this paper are available in the papers cited.

## References

[bib1.bib1] Apper E, Meymerit C, Bodin JC, Respondek F, Wagner A (2016). Effect of dietary supplementation with short-chain fructooligosaccharides in lactating sows and newly weaned piglets on reproductive performance of sows, immune response, and growth performance of piglets from birth to slaughter. Anim Res Nutr.

[bib1.bib2] Apolinário AC, de Lima Damasceno BP, de Macêdo Beltrão NE, Pessoa A, Converti A, da Silva JA (2014). Inulin-type fructans: A review on different aspects of biochemical and pharmaceutical technology. Carbohydr Polym.

[bib1.bib3] Babu US, Sommers K, Harrison LM, Balan KV (2012). Effects of fructooligosaccharide-inulin on *Salmonella*-killing and inflammatory gene expression in chicken macrophages. Vet Immunol Immunop.

[bib1.bib4] Baggio LL, Drucker DJ (2007). Biology of incretins: GLP-1 and GIP. Gastroenter.

[bib1.bib5] Barrow PA, Fuller R, Fabian P (1992). Probiotics for chickens. Probiotics: The scientific basis.

[bib1.bib6] Bergman EN (1990). Energy contributions of volatile fatty acidsfrom the gastrointestinal tract in various species. Physiol Rev.

[bib1.bib7] Blaut M (2002). Relationship of prebiotics and food to intestinal microflora. Eur J Nutr.

[bib1.bib8] Bogusławska-Tryk M, Piotrowska A, Burlikowska K (2012). Dietary fructans and their potential beneficial influence on health and performance parameters in broiler chickens. J Centr Eur Agricul.

[bib1.bib9] Bunce TJ, Howard MD, Kerley MS, Allee GL, Pace LW (1995). Protective effect of fructooligosaccharides in prevention of mortality and morbidity from infectious E. coli K: 88 challenge. J Anim Sci.

[bib1.bib10] Caetano BFR, de Moura NA, Almeida APS, Dias MC, Sivieri K, Barbisan LF (2016). Yacon (Smallanthus sonchifolius) as a Food Supplement: Health-Promoting Benefits of Fructooligosaccharides. Nutrients.

[bib1.bib11] Campbell JM, Fahey Jr GC, Wolf BW (1997). Selected indigestible oligosaccharides affect large bowel mass, cecal and fecal short-chain fatty acids, pH and microflora in rats. J Nutr.

[bib1.bib12] Chang M, Zhao Y, Qin G, Zhang X (2018). Fructo-Oligosaccharide Alleviates Soybean-Induced Anaphylaxis in Piglets by Modulating Gut Microbes. Front Microbiol.

[bib1.bib13] Choi KH, Namkrng H, Paid IK (1994). Effects of dietary fructooligosaccharide on the suppression of intestinal colonization of Salmonella typhimurium in broiler chickens. Kor J Anim Sci.

[bib1.bib14] Conway PL, Souffrant WB, Hagemeister H (1994). Function and regulation of the gastrointestinal microbiota of the pig. Digestive Physiology in Pigs.

[bib1.bib15] Crittenden RG, Playne MJ (1996). Production, properties and applications of foodgrade oligosaccharides. Trends Food Sci Tech.

[bib1.bib16] Cummings JH, Englyst HN (1995). Gastrointestinal effects of food carbohydrate. Am J Clin Nutr.

[bib1.bib17] De Filippo C, Cavalieri D, DiPaola M, Ramazzotti M, Poullet JB, Massart S, Collini S, Pieraccini G, Lionetti P (2010). Impact of diet in shaping gut microbiota revealed by a comparative study in children from Europe and rural Africa. P Natl Acad Sci USA.

[bib1.bib18] Delgado GT, Thomé R, Gabriel DL, Tamashiro WM, Pastore GM (2012). Yacon (*Smallanthus sonchifolius*)-derived fructooligosaccharides improves the immune parameters in the mouse. Nutr Res.

[bib1.bib19] Demir E, Sarica Ş, Özcan MA, Sui\, ¸Mez M (2010). The use of natural feed additives as alternatives for an antibiotic growth promoter in broiler diets. Brit Poultry Sci.

[bib1.bib20] Dominguez-Bello MG, Costello EK, Contreras M, Magris M, Hidalgo G, Fierer N, Knight R (2010). Delivery mode shapes the acquisition and structure of the initial microbiota across multiple body habitats in newborns. P Natl Acad Sci USA.

[bib1.bib21] Eberhard M, Hennig U, Kuhla S, Brunner RM, Kleessen B, Metges CC (2007). Effect of inulin supplementation on selected gastric, duodenal, and caecal microbiota and short chain fatty acid pattern in growing piglets. Arch Anim Nutr.

[bib1.bib22] Estrada A, Drew MD, Van Kessel A (2001). Effect of the dietary supplementation of fructooligosaccharides and *Bifidobacterium longum* to early-weaned pigs on performance and fecal bacterial populations. Can J Anim Sci.

[bib1.bib23] Flickinger EA, Van Loo J, Fahey Jr GC (2003). Nutritional responses to the presence of inulin and oligofructose in the diets of domesticated animals: A review. Crit Rev Food Sci Nutr.

[bib1.bib24] Fukata T, Sasai K, Miyamoto T, Baba E (1999). Inhibitory effects of competitive exclusion and fructo-oligosaccharide, singly and in combination, on *Salmonella* colonization of chicks. J Food Protect.

[bib1.bib25] Gibson GR, Roberfroid MB (1995). Dietary modulation of the human colonic microbiota: introducing the concept of prebiotics. J Nutr.

[bib1.bib26] Gibson GR, Roberfroid MB (2008). Handbook of Prebiotics.

[bib1.bib27] Gibson GR, Probert HM, Van Loo J, Rastall RA, Roberfroid MB (2004). Dietary modulation of the human colonic microbiota: updating the concept of prebiotics. Nutr Res Rev.

[bib1.bib28] He G, Baidoo SK, Yang Q, Golz D, Tungland B (2002). Evaluation of chicory inulin extracts as feed additive for early-weaned pigs. J Anim Sci.

[bib1.bib29] Herich R, Révayová V, Levkut M, Bomba A, Nemcová R, Guba P, Gancarčiková S (2002). The effect of Lactobacillus paracasei and Raftilose P95 upon the non-specific immune response of piglets. Food Agric Immunol.

[bib1.bib30] Hidaka H, Eida T, Takizawa T, Tokunaga T, Tashiro Y (1986). Effects of Fructooligosaccharides on Intestinal Flora and Human Health. Bif Micr.

[bib1.bib31] Houdijk JGM, Hartemink R, Verstegen MWA, Bosch M (2002). Effects of Dietary Non-Digestible Oligosaccharides on Microbial Characteristics of Ileal Chyme and Faeces in Weaner Pigs. Arch Anim Nutr.

[bib1.bib32] Inman CF, Harris C, Haverson K, Miller B (2005). The mucosal immune system of the neonatal piglet. Pig J.

[bib1.bib33] Jacela JY, De Rouchey JM, Tokach MD (2010). Feed additives for swine: Fact sheets – prebiotics and probiotics, and phytogenics. J Swine Health Prod.

[bib1.bib34] Jensen BB, Jørgensen H (1994). Effect of dietary fiber on microbial activity and microbial gas production in various regions of the gastrointestinal tract of pigs. Appl Environ Microb.

[bib1.bib35] Kaplan H, Hutkins RW (2000). Fermentation of Fructooligosaccharides by Lactic Acid Bacteria and Bifidobacteria. Appl Environ Microb.

[bib1.bib36] Khodambashi Emami N, Samie A, Rahmani HR, Ruiz-Feria CA (2012). The effect of peppermint essential oil and fructooligosaccharides, as alternatives to virginiamycin, on growth performance, digestibility, gut morphology and immune response of male broilers. Anim Feed Sci Tech.

[bib1.bib37] Kim HC, Park J, Kim M (2014). Gut Microbiota-Derived Short-Chain Fatty Acids, T Cells, and Inflammation. Immune Netw.

[bib1.bib38] Kleessen B, Hartman L, Blaut M (2001). Oligofructose and long-chain inulin: influence on the gut microbial ecology of rats associated with the human faecal flora. Brit J Nutr.

[bib1.bib39] Langen LV, Dieleman LA (2009). Prebiotics in chronic intestinal inflammation. Inflamm Bowel Dis.

[bib1.bib40] Le Bourgot C, Ferret-Bernard S, Le Normand L, Savary G, Menendez-Aparicio E, Blat S, Appert-Bossard E, Respondek F, Le Huërou-Luron I (2014). Maternal Short-Chain Fructooligosaccharide Supplementation Influences Intestinal Immune System Maturation in Piglets. PLoS ONE.

[bib1.bib41] Le Bourgot C, Ferret-Bernard S, Blat S, Apper E, Le Huërou-Luron I (2016). Short-chain fructooligosaccharide supplementation during gestation and lactation or after weaning differentially impacts pig growth and IgA response to influenza vaccination. J Funct Food.

[bib1.bib42] Le Bourgot C, Le Normand L, Formal M, Respondek F, Blat S, Apper E, Ferret-Bernard S, Le Huërou-Luron I (2017). Maternal short-chain fructo-oligosaccharide supplementation increases intestinal cytokine secretion, goblet cell number, butyrate concentration and *Lawsonia*
*intracellularis* humoral vaccine response in weaned pigs. Brit J Nutr.

[bib1.bib43] Le Bourgot C, Ferret-Bernard S, Apper E, Taminiau B, Cahu A, Le Normand L, Respondek F, Le Huërou-Luron I, Blat S (2019). Perinatal short-chain fructooligosaccharides program intestinal microbiota and improve enteroinsular axis function and inflammatory status in high-fat diet-fed adult pigs. Faseb J.

[bib1.bib44] Liu X, Zheng J, Zhou H (2011). TLRs as pharmacological targets for plant-derived compounds in infectious and inflammatory diseases. Int Immunopharmacol.

[bib1.bib45] Loh G, Eberhard M, Brunner RM, Hennig U, Kuhla S, Kleessen B, Metges CC (2006). Inulin Alters the Intestinal Microbiota and Short-Chain Fatty Acid Concentrations in Growing Pigs Regardless of Their Basal Diet${}^{1\text{–}3}$. J Nutr.

[bib1.bib46] Ohta A, Ohtsuki M, Baba S, Adachi T, Sakata T, Sakaguchi E (1995). Calcium and Magnesium Absorption from the Colon and Rectum Are Increased in Rats Fed Fructooligosaccharides. J Nutr.

[bib1.bib47] Macfarlane GT, Cummings JH (1999). Probiotics and prebiotics: can regulating the activities of intestinal bacteria benefit health?. Brit Med J.

[bib1.bib48] Micciche AC, Foley SL, Pavlidis HO, McIntyre DR, Ricke SC (2018). A Review of Prebiotics Against Salmonella in Poultry: Current and Future Potential for Microbiome Research Applications. Front Vet Sci.

[bib1.bib49] Mountzouris KC, Balaskas C, Fava F, Tuohy KM, Gibson GR, Fegeros K (2006). Profiling of composition and metabolic activities of the colonic microflora of growing pigs fed diets supplemented with prebiotic oligosaccharides. Anaerobe.

[bib1.bib50] Mussatto SI, Mancilha IM (2007). Nondigestible oligosaccharides: A review. Carb Pol.

[bib1.bib51] Penders J, Thijs C, Vink C, Stelma FF, Snijders B, Kummeling I, van den Brandt PA, Stobberingh EE (2006). Factors influencing the composition of the intestinal microbiota in early infancy. Pediatre.

[bib1.bib52] Peshev D, Van den Ende W (2014). Fructans: Prebiotics and immunomodulators. J Funct Foods.

[bib1.bib53] Rivero-Urgell M, Santamaria-Orleans A (2001). Oligosaccharides: application in infant food. Early Hum Dev.

[bib1.bib54] Roberfroid M, Gibson GR, Hoyles L (2010). Prebiotic effects: metabolic and health benefits. Brit J Nutr.

[bib1.bib55] Roberfroid MB (2005). Introducing inulin-type fructans. Brit J Nutr.

[bib1.bib56] Roberfroid MB, Delzenne NM (1998). Dietary fructans. Annu Rev Nutr.

[bib1.bib57] Roberfroid MB, Vanloo JAE, Gibson GR (1998). The bifidogenic nature of chicory inulin and its hydrolysis products. J Nutr.

[bib1.bib58] Roediger WEW (1980). Role of anaerobic bacteria in the metabolic welfare of the colonic mucosa in man. Gut.

[bib1.bib59] Sabater-Molina M, Larqué E, Torrella F, Zamora S (2009). Dietary fructooligosaccharides and potential benefits on health. J Physiol Biochem.

[bib1.bib60] Salminen S, Bouley C, Boutron-Ruault MC, Cummings JH (1998). Functional food science and gastrointestinal physiology and function. Brit J Nutr.

[bib1.bib61] Shang Y, Regassa A, Kim JH, Kim WK (2015). The effect of dietary fructooligosaccharide supplementation on growth performance, intestinal morphology, and immune responses in broiler chickens challenged with *Salmonella Enteritidis* lipopolysaccharides. Poultry Sci.

[bib1.bib62] Shim SB (2005). Effects of prebiotics, probiotics and synbiotics in the diet of young pigs (PhD Thesis).

[bib1.bib63] Shim SB, Williams IH, Verstegen MWA (2005). Effects of dietary fructo-oligosaccharide on villous height and disaccharidase activity of the small intestine, pH, VFA and ammonia concentrations in the large intestine of weaned pigs. Acta Agr Scand A-An.

[bib1.bib64] Singh RS, Singh RP, Kennedy JF (2016). Recent insights in enzymatic synthesis of fructooligosaccharides from inulin. Int J Biol Macromol.

[bib1.bib65] Sissons JW (1989). Potential of probiotic organisms to prevent diarrhea and promote digestion in farm animals: a review. J Sci Food Agr.

[bib1.bib66] Tolhurst G, Heffron H, Lam YS, Parker HE, Habib AM, Diakogiannaki E, Cameron J, Grosse J, Reimann F, Gribble FM (2012). Short-chain fatty acids stimulate glucagon-like peptide-1 secretion via the G-protein-coupled receptor FFAR2. Diabetes.

[bib1.bib67] Tsukahara T, Iwasaki Y, Nakayama K, Ushida K (2003). Stimulation of Butyrate Production in the Large Intestine of Weaning Piglets by Dietary Fructooligosaccharides and Its Influence on the Histological Variables of the Large Intestinal Mucosa. J Nutr Sci Vitaminol.

[bib1.bib68] Vandeplas S, Dubois Dauphin R, Beckers Y, Thonart P, Théwis A (2009). Salmonella in Chicken: Current and Developing Strategies To Reduce Contamination at Farm Level. J Food Protect.

[bib1.bib69] Van Loo J, Cummings J, Delzeene N, Englyst H (1999). Functional food properties of non-digestible oligosaccharides: a consensus report for the ENDO project (DGXII AIRII-CT94-1095). Brit J Nutr.

[bib1.bib70] Velez E, Castillo N, Mesón O, Grau A, Bonet MEB, Perdigón G (2013). Study of the effect exerted by fructo-oligosaccharides from yacon (Smallanthus sonchifolius) root flour in an intestinal infection model with Salmonella Typhimurium. Brit J Nutr.

[bib1.bib71] Verdonk JM, Shim SB, van Leeuwen P, Verstegen MW (2005). Application of inulin-type fructans in animal feed and pet food. Brit J Nutr.

[bib1.bib72] Wang X, Gibson GR (1993). Effects of the in vitro fermentation of oligofructose and inulin by bacteria growing on the human large intestine. J Appl Bacteriol.

[bib1.bib73] Williams J, Mallet S, Leconte M, Lessire M, Gabriel I (2009). The effects of fructo-oligosaccharides or whole wheat on the performance and digestive tract of broiler chickens. Brit Poultry Sci.

[bib1.bib74] Xu C, Chen X, Ji C, Ma Q, Hao K (2005). Study of the Application of Fructooligosaccharides in Piglets. Asian Austral J Anim.

[bib1.bib75] Xu ZR, Zou XT, Hu CH, Xia MS, Zhan XA, Wang MQ (2002). Effects of Dietary Fructooligosaccharide on Digestive Enzyme Activities, Intestinal Microflora and Morphology of Growing Pigs. Asian Austral J Anim.

[bib1.bib76] Xu ZR, Hu CH, Xia MS, Zhan XA, Wang MD (2003). Effects of dietary fructooligosaccharides on digestive enzyme activities, intestinal microflora and morphology of male broilers. Poultry Sci.

[bib1.bib77] Yang Y, Iji PA, Choct M (2009). Dietary modulation of gut microflora in broiler chickens: a review of the role of six kinds of alternatives to in-feed antibiotics. World's Poultry Sci J.

[bib1.bib78] Yason CV, Summers BA, KA (1987). Pathogenesis of rotavirus
infection in various age groups of chickens and turkeys: Pathology. Am J Vet Res.

[bib1.bib79] Yun JW (1996). Fructooligosaccharides-Occurrence, preparation, and application. Enzyme Microb Tech.

[bib1.bib80] Yu Wang MA, Tao Zeng MD, Shu-e Wang MA, Wei Wang MA, Qian Wang MA, Hong-Xia Yu MA (2010). Fructo-oligosaccharides enhance the mineral absorption and counteract the adverse effects of phytic acid in mice. Nutrition.

[bib1.bib81] Zhao PY, Wang JP, Kim IH (2013). Evaluation of dietary fructan supplementation on growth performance, nutrient digestibility, meat quality, fecal microbial flora, and fecal noxious gas emission in finishing pigs. J Anim Sci.

[bib1.bib82] Zhao W, Yuan M, Li P, Yan H, Zhang H, Liu J (2019). Short-chain fructo-oligosaccharides enhances intestinal barrier function by attenuating mucosa inflammation and altering colonic microbiota composition of weaning piglets. Ital J Anim Sci.

